# Pharmacy education and workforce: strategic recommendations based on expert consensus in Lebanon

**DOI:** 10.1186/s40545-022-00510-3

**Published:** 2023-01-02

**Authors:** Aline Hajj, Rony M. Zeenny, Hala Sacre, Marwan Akel, Chadia Haddad, Pascale Salameh

**Affiliations:** 1 INSPECT-LB (Institut National de Santé Publique, d’Épidémiologie Clinique Et de Toxicologie-Liban), Beirut, Lebanon; 2 Laboratoire de Pharmacologie, Pharmacie Clinique et Contrôle de Qualité Des Médicament, Faculty of Pharmacy, Saint Joseph University of Beirut, Beirut, Lebanon; 3 Faculté de Pharmacie, Université Laval, Québec, Canada; 4 Oncology Division, CHU de Québec Université Laval Research Center, Québec, Canada; 5 Department of Pharmacy, American University of Beirut Medical Center, Beirut, Lebanon; 6 School of Pharmacy, Lebanese International University, Beirut, Lebanon; 7 Research Department, Psychiatric Hospital of the Cross, Jal Eddib, Lebanon; 8 School of Health Sciences, Modern University for Business and Science, Beirut, Lebanon; 9 School of Medicine, Lebanese American University, Byblos, Lebanon; 10 Faculty of Pharmacy, Lebanese University, Hadath, Lebanon; 11 Department of Primary Care and Population Health, University of Nicosia Medical School, 2417 Nicosia, Cyprus

**Keywords:** Pharmacy, Education, Workforce, Strategy, Competency, Accreditation

## Abstract

Pharmacy in Lebanon has been taught for years, and the profession has known the golden ages in previous years. However, with the recent graduation of hundreds of pharmacists, without prior workforce planning, the oversupply of non-specialized pharmacists caused a mismatch with the needs of the market. The context of severe socioeconomic and sanitary crises has further exacerbated the situation, with hundreds of pharmacists leaving the country. A group of pharmacy experts joined to suggest strategic solutions to face such challenges, suggesting a clear strategy for education and the workforce, overarched by educational and professional values and based on six main pillars: (1) implement a national competency framework (including the core and specialized competency frameworks) to be used as a basis for licensure (colloquium); (2) implement a national pharmacy program accreditation, encompassing standards related to competencies adoption and assessment, curricula, teaching methods, research and innovation, instructors’ and preceptors’ skills, and experiential training; (3) organize training for students and early-career pharmacists; (4) optimize continuing education and implement continuous professional development, fostering innovation and specialization among working pharmacists; (5) develop and implement a pharmacy workforce strategy based on pharmacy intelligence, job market, and academic capacities; (6) develop and implement a legal framework for the above-mentioned pillars in collaboration with ministries and parliamentary commissions. Under the auspices of the relevant authorities, mainly the Order of Pharmacists of Lebanon and the Ministry of Education and Higher Education, the suggested strategy should be discussed and implemented for a better future for the pharmacy profession.

## Background

Pharmacy education in Lebanon dates back to 1871 when the first school of pharmacy was created at the American University of Beirut (AUB), but closed its doors in 1977 [1]. A few years later, in 1883, Saint Joseph University (USJ) started teaching pharmacy [2]. It was later followed by four others: The Lebanese University (1983), Beirut Arab University (1986), Lebanese American University (1999), and Lebanese International University (2004).

There are currently five universities teaching pharmacy in Lebanon (one public [3] and four private) [4–7]. Two universities teach according to the European system, two according to the American system, and one follows the Canadian system. A minimum of 5 years is mandatory to be able to practice pharmacy in Lebanon, including 12 months of training at any pharmaceutical institution under the preceptorship of a pharmacist. Pharmacy education programs grant a Bachelor of Science in Pharmacy (BS Pharm) degree (given per se by all universities, except for USJ, where the Doctor of Pharmacy (PharmD) degree is entry-level), while additional degrees are elective, such as PharmD or master’s degrees, which enroll around 50% of BS graduates. Health and educational authorities rarely regulate pharmacy education and training, which vary according to educational institutions, since existing laws only address the duration of undergraduate pharmacy studies and training. In the absence of official recognition of credentials [8], universities offer postgraduate programs randomly. Hence, the current situation of pharmacy practice is chaotic due to a lack of planning for pharmaceutical education and workforce, and rules for the recruitment of pharmacists depend on institutions, while offer and demand control the job market.

The Order of Pharmacists of Lebanon (OPL, the official pharmacists’ association in Lebanon) has endeavored to establish a minimum of 6 years of pharmacy education as entry-level, a suggestion that has not been legally approved yet, keeping the minimum of 5 years in effect. Moreover, residency and Ph.D. remain uncommon pathways (< 10%), as they are considered mainly by those intending to work in academia/research.

The book chapter “Pharmacy Education, Practice, and Research in Lebanon” presented an elaborated background assessment of pharmacy in Lebanon [9]. It showed that, for quality assurance, all universities have a different internationally recognized accreditation body. Also, they all base their education on competencies adopted by these accreditation agencies in the absence of an officially recognized competency framework at the national level. This discrepancy is potentially associated with graduating pharmacists with heterogeneous skills that may not necessarily meet the Lebanese job market needs [9].

The five universities graduate around 500 non-specialized pharmacists per year, added to those coming from outside Lebanon (between 50 and 100 per year). This number is considered one of the highest in the world, particularly for the BS Pharm (17.52/10,000 inhabitants up till the end of 2017 [10]), while the country lacks national policies that set the number of graduates per school according to the local needs. There are no precise data about the employability of graduates, but academic surveys demonstrate a decline in work opportunities over time [11].

The oversupply problem escalated starting in October 2019, as political turmoil and the pandemic deepened the socioeconomic crisis to an unprecedented level. Indeed, this oversupply has led to unemployment during COVID-19 and the economic crisis, as many pharmaceutical companies closed their or downsized due to the market shrinking and financial difficulties, thus affecting the job market size and decreasing opportunities for innovative practice and research support. Furthermore, there are no exact figures for how many Lebanese have fled the country since October 2019; nevertheless, it is known that out of 3400 community pharmacies, around 400 have shut down, and 70% of pharmacy graduates are attempting to leave Lebanon [12]. Many pharmacists (early graduates and seniors) are trying to emigrate, in alignment with the whole health workforce that is grasping any opportunity abroad (Gulf countries, Europe, and North America) [13]. There are no policies or enforced legislation related to pharmacy education and the workforce to face this disastrous situation; the absence of comprehensive strategies even hampers the application of existing laws, opening the door to a chaotic situation. Overall, improving the quality of pharmacy education is a moral obligation and optimizing the workforce adequacy to local needs is a must, despite logistic, legal, cultural, and practical difficulties [14].

To guide countries in their challenging endeavors, the International Pharmaceutical Federation (FIP) issued a new core competency framework for pharmacy (the Global Competency Framework—GbCF) [15, 16] and a Global Advanced Framework (GADF) [17], followed by a framework for emergency readiness [18]. It also generated a document about Development Goals for pharmacy education, practice, and research [19]. The FIP documents are aligned with and complement the UN SDG [20] for health and education (SDG3 and SDG4). These references would guide countries in optimizing pharmacy at the national, early-career, and advanced pharmacy levels.

## Objectives

This document sought to assess the need for a strategy related to pharmaceutical education and workforce to identify the strengths and weaknesses of pharmaceutical education and workforce in Lebanon based on the current situation in the country. It also aimed to suggest a strategy to improve the quality of pharmacy education in Lebanon while considering essential learning outcomes, competency frameworks, and individualized development needs. Then, evidence-based expected decisions would be implemented under the leadership and supervision of involved pharmaceutical authorities and with the guidance of international instances.

## Methods

Recently, six experts (authors of the manuscript) joined efforts to suggest a clear strategy to face the current challenges of pharmacy in Lebanon; five are members of the OPL scientific committee, four are also academic pharmacists (two mid-career and two seniors), and one is a managing director at the OPL. All authors have been involved in research on various aspects of pharmacy education, workforce, and practice.

The project started with the assessment of previous works related to pharmacy education and workforce. Documents, including laws, regulations, white papers, and published articles, were sought in the archives of the OPL and through a thorough literature review. The authors conducted GAP and SWOC analyses and then suggested the vision, values, strategic objectives, and strategy implementation guidance. A Delphi technique using several rounds was applied until a consensus was reached on the entire document.

Finally, the current strategy was endorsed by the OPL higher administration and submitted to the relevant authorities (Ministry of Public Health and Ministry of Education and Higher Education) for approval as a part of the National Pharmaceutical Strategy in Lebanon and its implementation plan [21].

## Results

### GAP analysis assessment

Since 2016, the OPL scientific committee, which included academic pharmacists, has been working on assessment documents related to pharmacy education, workforce, and practice. The related literature review is summarized in Table 1, detailing problematic aspects of the profession, from initial education to continuous professional development, workforce planning, research promotion, and innovative practices. However, the suggested solutions have not been implemented for political reasons.

**Table 1:** Literature review related to the pharmacy in Lebanon

Field	Sub-domain	References
Initial education	Core competencies initial suggestion	[48]
	Core competencies framework validation	[49]
	Core competencies assessment	[50]
	Preceptors’ skills and competencies framework	[51]
	Specialized competencies suggestion	[52]
	Interprofessional education experience and suggestions	[53–55]
	Computer literacy scale validation among pharmacists	[56]
Continuing education (CE)	Continuing education assessment	[57]
	Continuing education preference	[58, 59]
	Continuing education value and motivation	[60]
Job market and workforce situation	Workforce oversupply and future projections	[10]
	Mismatch between education and labor market	[61]
	Governance solutions for workforce problems	[62]
	Pharmacy specialties recognition (credentialing)	[63]
	Early career training in Lebanon and benchmarking towards the FIP Global Competency Framework	[64]
	Empathy towards patients among pharmacists	[65]
	Quality of life of pharmacists	[66]
	Burnout among community pharmacists	[67, 68]
	Financial situation analysis of pharmacists	[69]
	Community pharmacists’ readiness for COVID-19	[70]
	Physical Health of pharmacists	[71]
Unapproved suggested laws and decisions	Legal documents suggestions by the Lebanese Order of Pharmacists: Clinical pharmacy law; *Numerus clausus* law, Pharmacy education prolongation, credentials recognition and Early career training	[72]
	Licensure assessment improvement (colloquium)	[72]
	Experiential education organization	[73]
Ready-to-apply educational project	Standards for pharmacy education accreditation & Guidance for pharmacy programs accreditation	[74]
Practice	Good Pharmacy Practice standards suggestion	[75]
	Good Pharmacy Practice assessment	[76]
	Medication safety assessment	[77–79]
	Electronic patient profile suggestion	[80]
	Pharmacist–physician collaborative care	[81]
	Societal perspective of pharmacy	[82]
	Primary health care and community pharmacy	[83]
	Primary care vision for pharmacists	[84]
	Seeking care from pharmacists	[85]
Research	Researcher pharmacists’ competencies	[86]
	Role of a professional organization in research	[87]

A detailed GAP analysis was later conducted for pharmacy education, workforce, practice, and sciences, through a benchmarking exercise with the FIP 2020 Development Goals that bring together workforce/education [W], practice [P], and science [S] in a transformative framework [22]. The cited document stated that in the short term, projects already developed by the OPL and suggested to the relevant authorities should be implemented immediately, e.g., pharmacy curricula reforms based on updated accreditation standards, the recognition of postgraduate training and pharmacy specialties, the organization of pharmacy education and practice, and the assessment of advanced competencies in different pharmacy areas. In parallel, existing policies, strategies, and regulations should be updated. Cultural changes involving closer ties between education and practice are pivotal to conducting the appropriate reform of the pharmacy profession; overall, efforts are necessary to bridge the gap between the current situation of the pharmacy profession in Lebanon and optimal practice. The recommended initiatives are part of the currently suggested education and workforce strategy.

Of note, complementary aspects related to research and practice have already been tackled. In the medium term, a national research strategy was recommended, including early-career scientists, mentoring, and validation of assessment tools [22]. A recently developed Pharmaceutical Research Strategy was suggested subsequently [23].

As for practice, digitalizing the patient profile, reporting medication safety events, and updating the pharmacists’ database through appropriate platforms were steps deemed essential toward modernizing pharmacy, as recommended by the recently developed national pharmaceutical strategy [21].

### SWOC analysis related to pharmacy education and workforce

Based on the conducted GAP assessment and the related literature review, the SWOC analysis related to the education and workforce situation is presented in Table 2. A consensus was reached regarding all aspects, including the strengths of the profession and its educational institutions, the mismatch between the educational outcomes and the job market, the effect of the current crisis on education and workforce, the need for national and international collaborations in education and research, and the need for a political will to adopt suggested decisions and laws based on updated assessment activities. Strengths should be consolidated, opportunities seized, weaknesses addressed, and challenges faced through appropriate initiatives.

**Table 2:** SWOC analysis of the workforce education and job market

Internal factors	
Strengths	Weaknesses
Education • Pharmacy is a profession that attracts good students with a low attrition rate • Admission criteria take grades into account; select the best through the entrance examination • Time to graduation is acceptable for more than 90% of students • All pharmacy programs are accredited by international authorities, which is a quality label • International accreditation of programs is an opportunity for continuous improvement of pharmacy programs • There is no deficiency in community pharmacy distribution, as urban and rural regions are well served • Continuing education is mandatory by the OPL according to the law • Interprofessional education has been implemented in some universities • Several universities have established residency and PhD programs in collaboration with international partners • Universities graduate students of good quality, accepted in many international positions • Interprofessional education is being applied in some universities, paving the way to interprofessional collaborative practice Workforce • A decree has been issued to limit the position of medical representatives to pharmacists to increase employment opportunities for pharmacists; however, it is not yet applied • The clinical pharmacy law has been suggested but has been awaiting approval for more than 20 years • Universities have highly qualified academics who can upgrade curricula based on new competencies frameworks	Education • There is no legal framework for pharmacy education • There is no national pharmacy workforce strategy • Collaboration between universities is minimal • There is no clear guidance for undergraduate and postgraduate training • There is an oversupply of non-specialized pharmacists • Curricula need to be updated according to the latest international recommendations • New sections related to public health and soft skills should be added to curricula in some universities • There is no official up-to-date core competency framework • There is no official specialized competency framework • Specialized competencies are not assessed • There is no adequate competency-based licensure examination • There is no national accreditation agency, thus no homogeneous conditions for graduation from different universities • There is no national research strategy Workforce •There is an oversupply of non-specialized pharmacists • There are no exact figures about the employability of graduates • The number of pharmacists who graduated from outside Lebanon is relatively high • Pharmacy specialties are not formally recognized • There is no early career training program • There are no regulations for a pharmacy support workforce (pharmacy technicians) • Market needs for specialties are not assessed • Some pharmacists are resistant to changes related to continuing education and other new concepts (digitalization, accreditation, and others) • There is no framework for interprofessional collaborations, which causes overlapping education, practice, and competing interests

External factors	
Opportunities	Challenges/threats
• Policy suggestions related to pharmacy education and the workforce are waiting to be approved • The MOPH is working on a comprehensive national strategy for health • The OPL is leading on a national pharmaceutical strategy in collaboration with other stakeholders (ministries, WHO, pharmaceutical sector, and other healthcare professionals) • Ongoing strategies that are being prepared should be complemented by a pharmacy workforce strategy • The pharmacy sector benefits from collaborations with international agencies (FIP, WHO, CIOPF, etc.), which would optimize the exchange of ideas and capacity building • International non-governmental organizations are offering positions for pharmacists, in particular, to organize medication and pharmaceutical services delivery for underprivileged populations	Education • Severe socioeconomic and ongoing sanitary crises are affecting education conditions • Experiential education during severe economic and sanitary crises is not immersive, as it is done virtually • Online education is given in difficult circumstances (shortage of power and internet supply), which might jeopardize its quality • Highly motivated young pharmacy graduates have international aspirations, which might increase emigration Workforce • Severe socioeconomic and ongoing sanitary crises contribute to the deterioration of financial conditions • The professional situation of all pharmacists is currently disastrous (extreme financial difficulties) • Many pharmaceutical companies are closing their local offices • Many working pharmacists are leaving the country for better opportunities • Medication shortage is very severe, putting at risk the health of professionals and patients • Interprofessional education and collaborative practice are cultural challenges; healthcare professionals are reluctant to collaborate • The presence of refugees in very high numbers is pressuring the scarce resources of healthcare and educational systems

*MOPH* Ministry of Public Health, *OPL* Order of Pharmacists of Lebanon, *WHO* World Health Organization, *FIP* International Pharmaceutical Federation, *CIOPF* Conférence Internationale des Ordres de Pharmaciens Francophones

### Suggested vision

While pharmacy education should serve the ever-changing role of the pharmacist in different fields of specialty (e.g., community, hospital, clinical, industrial pharmacy, and clinical biology), the following vision was suggested: “pharmacy education in Lebanon should comply with the highest international, regional, and national standards to graduate practice-ready pharmacists in different specialties and provide safe, compassionate, and high-quality care to improve the health and well-being of the people in Lebanon”.

### Suggested values

**Fig. 1:**
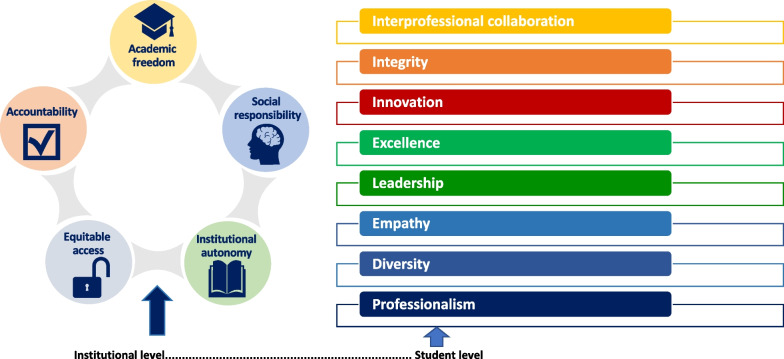
Pharmacy education values at institutional and individual levels

Higher education encompasses several concepts and should optimize instructors’ teaching and students’ learning, the outcome being student achievement, engagement, and well-being, leading to higher employability and success in life. Factors related to teaching and learning optimization involve teaching methods, curriculum planning, and assessment based on professional competencies (when possible), a positive climate for learning, building practice excellence, professional leadership, and community engagement in learning. These factors would be applied based on international human rights law, UNESCO (United Nations Educational, Scientific and Cultural Organization) instruments for ethics and values, and related civil society statements; thus, five core values are collectively identified in higher education and are crucial to be adopted in higher education institutions [24]. Moreover, taking specific pharmacy education values into account is also a must; these values should be taught to pharmacy students [25]. They should also be tightly aligned with the United Nations Sustainable Development Goals [26] and the FIP Development Goals [27]. Based on these concepts, 14 values have been suggested, five at the institutional level (equitable access, accountability, institutional autonomy, academic freedom, and social responsibility) and nine at the student level (interprofessional collaboration, integrity, innovation, discovery (research), excellence, leadership, empathy, diversity, and professionalism) (Fig. 1).

### Pillars and strategic objectives

**Fig. 2:**
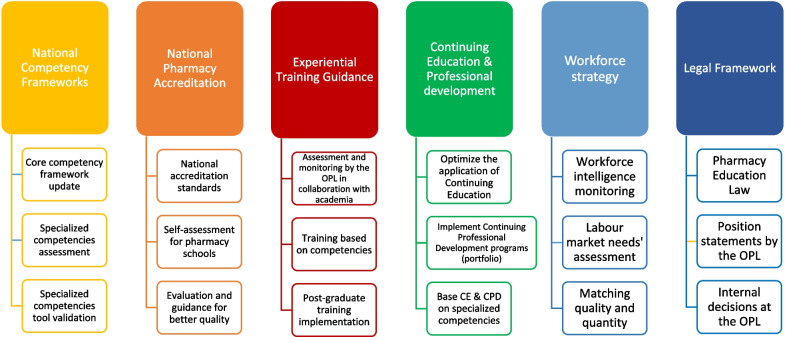
Pillars and objectives for pharmacy education and workforce

Several strategic objectives could be developed and categorized into six main pillars based on the literature search, the GAP/SWOC analyses, and the suggested vision and values, to optimize pharmacy education and workforce in Lebanon. Policy dialogues would be necessary to derive activities and prioritize and distribute tasks to reach every objective; the whole endeavor should be continuously assessed through appropriate key performance indicators. The main pillars and objectives were as follows (Fig. 2):

1. Implement a national competency framework (including the core and specialized competency frameworks) to be used as a basis for licensure (colloquium);
2. Implement a national pharmacy program accreditation, encompassing standards related to the adoption and assessment of competencies, curricula, teaching methods, research and innovation, instructors’ and preceptors’ skills, and experiential training;
3. Organize training for students and early-career pharmacists;
4. Optimize continuing education and implement continuous professional development, fostering innovation and optimizing specialization among working pharmacists;
5. Develop and implement a pharmacy workforce strategy based on pharmacy intelligence, job market, and academic capacities;
6. Develop and implement a legal framework for the above-mentioned pillars in collaboration with ministries and parliamentary commissions.

### Strategy implementation guidance

Many stakeholders are involved in the application of the strategy, including academia, the Ministry of Education and Higher Education (MEHE), the Ministry of Public Health (MOPH), professional organizations (OPL, the Syndicates of Clinical Biology, and others), and the pharmaceutical sector (local industries, multinational companies, and importers). The help of international instances, such as the WHO and the FIP, would also be valuable. The suggested strategy should be implemented in Lebanon as soon as possible; strategy implementation should go hand in hand with policy development/update.

A task force involving experienced pharmacists and professionals from all stakeholders should start the work and move forward with the advocacy of the strategy, develop an implementation plan, and showcase the short-term and long-term benefits this strategy will have on education and practice. Success stories from around the globe may serve as proof of how this transformation leads to the desired outcomes. Collaboration among stakeholders is a must to save the endangered profession [28]. The adoption and implementation of this strategy are expected to take effort and time, particularly since many barriers are restraining the progress of any initiative, whether the legal framework is available or not. Lastly, this document could serve as a case study for countries with similar problems and help them build their own strategy.

## Discussion

In this document, we were able to develop a comprehensive strategy for pharmacy education and the workforce in Lebanon. To optimize pharmacy education and workforce, authorities and relevant stakeholders should coordinate efforts to implement initiatives based on the available assessment, international guidance documents, and the currently suggested strategy. Joint efforts are also expected to bridge the gap between the current challenging situation of the profession in Lebanon and its potential future development for the best of the pharmacist and optimal patient health.

Although the suggested strategy and its implementation plan are evidence-based, straightforward, and expected to advance the profession in Lebanon and scale it up to international levels, many barriers would hamper this endeavor. The most obvious roadblock is the country’s economic and sanitary crises that have heavy consequences on the pharmaceutical sector: severe medications shortage affecting essential medications [29], chaotic substandard medications market due to insufficient monitoring regulations [30], pharmacists’ financial difficulties, downsizing (even relocating or closing) of multinational companies local offices, emigration of many pharmacists (including educators, researchers, junior and senior practitioners…) [31]. These factors are creating a context of the hesitancy of good students to join the profession, a doubtful pharmacy education quality due to online-related problems (e.g., power shortages and internet unreliability) [32], challenging experiential education [33], and a lack of continuing education activities in times of crises. At the workforce level, job opportunities are gradually lost. The implementation plan should account for these substantial challenges and find innovative ways to overcome them [34, 35].

The second barrier is the political complexity of the country: the multiplicity of cultures co-living in Lebanon, the wars that had shredded it for 15 years [36], and the economic aftermath of the war [37] led to the instauration of a system based on consensus between components and short-term reactions to uprising problems, without long-term strategies that could sustain health and develop the country. A difficult-to-achieve culture change is necessary through insightful leadership [38], closing the door to political interference and sectorial intrusion, opening minds to strategic thinking, and forecasting solutions to different scenarios. Policy dialogues, legal decisions, and implementation of actions through appropriate governance structures and institutional organization [39] would then lead the professional components, including education and the workforce, toward their goals.

The third expected hurdle is the interest of university administrations: a homogenization of competencies and standards might be understood as an attack on institutional autonomy [40, 41] and an attempt to reduce the number of graduates in the private sector based on possible sectarian and financial interests among higher institutions [42, 43]. The resistance of involved instructors can also be expected, particularly among seniors, and should be overcome through dialogue and appropriate training [44]. The good of the pharmacists’ community should have moral priority over individualistic interests in the particular situation of a profession at risk of annihilation.

Last but not least, the conflict of healthcare professions is the fourth barrier in the absence of clear interprofessional boundaries and fields of collaboration [45]. Despite the availability of many laws that regulate health professions [46], their application is not always enforced in Lebanon, and some legislations might need to be updated. Thus, revising and re-enforcing the current laws, including mandatory interprofessional education in curricula and legislation related to interprofessional collaboration in integrative healthcare [47], would define conflicting interests, decrease the tension, and smoothen healthcare practices, knowing that all are thriving to serve the patient.

Overcoming the described barriers would require joining efforts of all stakeholders, with policy dialogue for every strategic objective, similar to what was done for the national pharmaceutical strategy [21]. The leadership of the OPL on this matter is expected to be of utmost importance.

## Conclusion

The suggested document should be discussed and implemented for a better future of the pharmacy profession under the auspices of the involved authorities, mainly the Order of Pharmacists of Lebanon and the Ministry of Education and Higher Education. Uniting the efforts of stakeholders would serve to realize the vision of a thriving pharmacy education and workforce, leading to better practices and, ultimately, optimal patient health.

## Data Availability

Not applicable.

## Abbreviations

BS Pharm: Bachelor of Science in Pharmacy
CIOPF: Conférence Internationale des Ordres de Pharmaciens Francophones
DGs: Development goals
FIP: International Pharmaceutical Federation
GADF: Global Advanced Framework
GbCF: Global Competency Framework
MEHE: Ministry of Education and Higher Education
MOPH: Ministry of Public Health
OPL: Order of Pharmacists of Lebanon
PharmD: Doctor of Pharmacy
SWOC: Strengths, weaknesses, opportunities, challenges/threats
UNESCO: United Nations Educational, Scientific and Cultural Organization
WHO: World Health Organization

## References

[1] Khachan V, Saab Y, Sadik F (2010). Pharmacy education in Lebanon. Curr Pharm Teach Learn.

[2] Université Saint-Joseph. Faculté de Pharmacie, Historique. 2021 [cited 2021 March 11]; Available from: https://usj.edu.lb/fp/.

[3] Pell G (2012). Is short-term remediation after OSCE failure sustained? A retrospective analysis of the longitudinal attainment of underperforming students in OSCE assessments. Med Teach.

[4] Guerrasio J, Garrity MJ, Aagaard EM (2014). Learner deficits and academic outcomes of medical students, residents, fellows, and attending physicians referred to a remediation program, 2006–2012. Acad Med.

[5] Wiese, J. A curriculum to observe underachieving students and give assisted remediation (cougar). J Gen Intern Med. 2000. Blackwell Science Inc 350 Main St, Malden, MA 02148 USA.

[6] Segal SS (1999). The academic support program at the University of Michigan School of Medicine. Acad Med.

[7] Frellsen SL (2008). Medical school policies regarding struggling medical students during the internal medicine clerkships: results of a national survey. Acad Med.

[8] Hallit S (2019). Credentialing and recognition of pharmacy specializations: the Lebanese order of pharmacists initiative. ACCP Int Clin Pharm.

[9] Hajj A, Sacre H, Salameh P. Pharmacy Education, Practice, and Research in Lebanon, in Handbook of Medical and Health Sciences in Developing Countries: Education, Practice, and Research. 2021, Springer, Berlin.

[10] Hallit S (2019). Projecting the future size of the Lebanese pharmacy workforce: forecasts until the year 2050. Int J Pharm Pract.

[11] Nassar E (2022). A pilot assessment of the career and job satisfaction of the pharmaceutical workforce in Lebanon. J Pharmaceut Policy Pract.

[12] Karam Z. Driven by despair, Lebanese pharmacist looks to life abroad. 2021 [cited 2022 March 27]; Available from: https://apnews.com/article/beirut-europe-middle-east-lebanon-immigration-c24d8997337510786121eac87eec1bd6.

[13] Yassine H. Hundreds Of Doctors And Nurses Are Emigrating From Lebanon. 2020 [cited 2022 March 27]; Available from: https://www.the961.com/doctors-nurses-emigrating-lebanon/#:~:text=Since%20late%202019%2C%20the%20instability,are%20leaving%20for%20better%20opportunities.

[14] Al-Ghananeem AM (2018). A call to action to transform pharmacy education and practice in the Arab World. Am J Pharm Educ.

[15] International Pharmaceutical Federation (FIP). Pharmacy Education Taskforce: A Global Competency Framework. 2012 [cited 2020 July 24]; Available from: https://www.fip.org/files/fip/PharmacyEducation/GbCF_v1.pdf.

[16] International Pharmaceutical Federation (FIP). The FIP Development Goals: Transforming global pharmacy. 2020 [cited 2021 August 21]; Available from: https://www.fip.org/file/4793.

[17] International Pharmaceutical Federation (FIP). FIP Global Advanced Development Framework (GADF): Supporting the advancement of the profession. 2019 [cited 2022 March 27]; Available from: https://www.fip.org/file/4331.

[18] Grenier S et al. FIP Global Humanitarian Competency Framework—GbHCF, Supporting pharmacists and the pharmaceutical workforce in a humanitarian arena. 2022 [cited 2022 April 1]; Available from: https://gsspharmacie.wordpress.com/2022/01/14/fip-global-humanitarian-competency-framework-gbhcf-supporting-pharmacists-and-the-pharmaceutical-workforce-in-a-humanitarian-arena/.

[19] International Pharmaceutical Federation (FIP) FIP Development Goals. 2021 [cited 2022 March 27]; Available from: https://www.fip.org/search?page=fip-development-goals.

[20] United Nations Development Programme (UNDP) The SDGs in action. 2022 [cited 2022 April 1]; Available from: https://www.undp.org/sustainable-development-goals?utm_source=EN&utm_medium=GSR&utm_content=US_UNDP_PaidSearch_Brand_English&utm_campaign=CENTRAL&c_src=CENTRAL&c_src2=GSR&gclid=CjwKCAjwopWSBhB6EiwAjxmqDSenO0f7aM3Rd2k3QantJ_uMmuewlYhjwapShLafZCl9NiFbs0gbPBoCa3kQAvD_BwE.

[21] Sacre H, et al. Towards a national pharmaceutical strategy in Lebanon: a commitment and a call to action to ensure access to quality and safe medications in Lebanon for all. The Order of Pharmacists of Lebanon, 2022.

[22] Sacre H (2021). Pharmacy education, workforce, practice, and sciences in Lebanon: benchmarking with the FIP Development Goals. Pharm Educ.

[23] Akel M (2022). Developing a national pharmaceutical research strategy in Lebanon: opportunities to bridge the gaps and reach the goals. J Pharmaceut Policy Pract.

[24] Scholars at Risk. Promoting Higher Education Values: a guide for discussion. 2017 [cited 2022 March 27]; Available from: https://www.scholarsatrisk.org/wp-content/uploads/2018/08/SAR_Promoting-Higher-Education-Values-Guide.pdf.

[25] Binghamton University State University of New York. Mission, Vision and Core values. 2022 [cited 2022 March 27]; Available from: https://www.binghamton.edu/pharmacy-and-pharmaceutical-sciences/about/mission-vision.html.

[26] Sachs JD (2012). From millennium development goals to sustainable development goals. Lancet.

[27] International Pharmaceutical Federation (FIP). The FIP Development Goals report 2021. 2022 [cited 2022 March 27]; Available from: https://farmaciavirtuale.it/wp-content/uploads/2022/01/3116-Development-Goals-report-2021-Fonte-FIP.pdf.

[28] Alameddine M (2020). A profession in danger: Stakeholders' perspectives on supporting the pharmacy profession in Lebanon. PLoS ONE.

[29] Das M (2021). Lebanon faces critical shortage of drugs. Lancet Oncol.

[30] Hobeika E (2020). Are antibiotics substandard in Lebanon? Quantification of active pharmaceutical ingredients between brand and generics of selected antibiotics. BMC Pharmacol Toxicol.

[31] Shallal A (2021). Lebanon is losing its front line. J Glob Health.

[32] Khaddage F, Fayad R, Moussallem I. Online Learning and the Role of Technologies During COVID19 Pandemic “Higher Education Lebanon Case” in Proceedings of EdMedia + Innovate Learning. 2020, Association for the Advancement of Computing in Education (AACE): The Netherlands. p. 623–630.

[33] Christian D, McCarty D, Brown C (2021). Experiential education during the COVID-19 pandemic: a reflective process. J Construct Psychol.

[34] Schalock R. Leadership Guide for Today's Disabilities Organizations: Overcoming Challenges and Making Change Happen. 2012, Baltimore.

[35] Luppi E (2021). Overcoming challenges and boundaries through the innovation of university learning and teaching practices. The promise of higher education.

[36] Tas Y (2020). Lebanon: confessionalism and the problem of divided loyalties. Routledge handbook on the governance of religious diversity.

[37] Baumann H (2019). The causes, nature, and effect of the current crisis of Lebanese capitalism. National Ethnic Polit.

[38] Stel N. Entrepreneurship and innovation in a hybrid political order: The case of Lebanon. 2012 [cited 2022 March 27]; Available from: https://ideas.repec.org/p/unm/unumer/2012078.html.

[39] El Haddad P, Auffret J, Grishina O (2020). Public administration reform in Lebanon from the leadership perspective. Res Sci Gestion.

[40] Salto D (2018). Quality assurance through accreditation: when resistance meets over-compliance. High Educ Q.

[41] Campos B. The balance between higher education autonomy and public quality assurance: development of the Portuguese system for teacher education accreditation. Education Policy Analysis Archives. 2004; 12(73).

[42] Baytiyeh H (2017). Has the educational system in Lebanon contributed to the growing sectarian divisions?. Educ Urban Soc.

[43] Nahas C (2011). Financing and political economy of higher education in Lebanon. Pospects.

[44] Abouelenein Y (2016). Training needs for faculty members: towards achieving quality of university education in the light of technological innovations. Educ Res Rev.

[45] Sexton M, Orchard C (2016). Understanding healthcare professionals' self-efficacy to resolve interprofessional conflict. J Interprof Care.

[46] Ministry of Public Health. Laws and Regulations. 2022 [cited 2022 April 1]; Available from: https://moph.gov.lb/en/laws

[47] Chung VC (2012). Organizational determinants of interprofessional collaboration in integrative health care: systematic review of qualitative studies. PLoS ONE.

[48] Sacre H et al. Developing core competencies for pharmacy graduates: the Lebanese experience. J Pharm Pract. 2020: 897190020966195. 10.1177/0897190020966195 33084476

[49] Hajj A (2021). Lebanese pharmacy core competencies framework: tool validation for self-declared assessment. Int J Pharm Pract.

[50] Zeenny RM (2021). Descriptive assessment of graduates' perceptions of pharmacy-related competencies based on the Lebanese pharmacy core competencies framework. Pharm Pract (Granada).

[51] Zeitoun A (2020). Clinical preceptor competencies for a better pharmacy education: a suggested framework for Lebanon. J Pharm Policy Pract.

[52] Sacre H (2020). Upgrading pharmacy education to produce practice-ready pharmacists in Lebanon. Pharm Educ.

[53] Farra A (2018). Implementing an interprofessional education programme in Lebanon: overcoming challenges. Eastern Mediterr Health J..

[54] Hajj A (2019). Assessment of knowledge, attitude and practice among community pharmacists towards dental care: a national cross sectional survey. Saudi Pharm J.

[55] Hajj A (2021). Assessment of drug-prescribing perception and practice among dental care providers: a cross-sectional Lebanese study. Pharm Pract (Granada).

[56] Hallit S (2020). Lebanese pharmacists' confidence and self-perceptions of computer literacy: scale validation and correlates. J Pharm Policy Pract.

[57] Sacre H (2019). Mandatory continuing education for pharmacists in a developing country: assessment of a three-year cycle. Pharm Pract (Granada).

[58] Iskandar K (2018). Assessing continuing education practices and preferences amongst Lebanese Hospital Pharmacists. Pharm Pract (Granada).

[59] Sacre H (2019). Attitudes of Lebanese pharmacists towards online and live continuing education sessions. Pharm Pract (Granada).

[60] Tawil S (2020). Pharmacists and continuing education: a cross-sectional observational study of value and motivation. Int J Pharm Pract.

[61] Alameddine M, BouKarroum K, Hijazi MA (2019). Upscaling the pharmacy profession in Lebanon: workforce distribution and key improvement opportunities. Hum Resour Health.

[62] Sacre H (2019). The pharmacy profession in a developing country: challenges and suggested governance solutions in Lebanon. J Res Pharm Pract.

[63] Hallit S et al. Credentialing and recognition of pharmacy specializations: the Lebanese order of pharmacists initiative. ACCP International Clinical Pharmacists, 2019.

[64] Hajj A (2022). The Lebanese experience for early career development: Bridging the gap to reach the International Pharmaceutical Federation (FIP) Global Competency Framework. Pharm Educ.

[65] Hobeika E (2020). Factors associated with empathy among community pharmacists in Lebanon. J Pharm Policy Pract.

[66] Sacre H (2019). Factors associated with quality of life among community pharmacists in Lebanon: results of a cross-sectional study. Pharm Pract (Granada).

[67] Youssef D (2021). Prevalence and risk factors of burnout among Lebanese community pharmacists in the era of COVID-19 pandemic: results from the first national cross-sectional survey. J Pharmaceut Policy Pract.

[68] Alameddine M, Bou-Karroum K, Hijazi MA (2022). A national study on the resilience of community pharmacists in Lebanon: a cross-sectional survey. J Pharm Policy Pract.

[69] Hallit S (2017). Situation analysis of community pharmacy owners in Lebanon. Pharm Pract (Granada).

[70] Zeenny RM (2021). A cross-sectional survey on community pharmacists readiness to fight COVID-19 in a developing country: knowledge, attitude, and practice in Lebanon. J Pharm Policy Pract.

[71] Barake S (2021). The health status of Lebanese community pharmacists: prevalence of poor lifestyle behaviors and chronic conditions. Saudi Pharm J.

[72] Order of Pharmacists of Lebanon (OPL). OPL deliverables 2015–2018. Summary of a Mandate. Clinical pharmacy law; Numerus Clausus law. Pharmacy education prolongation. Early career decision.; Available from: https://opl.org.lb/newdesign/pdf/OPL%20Deliverables.pdf.

[73] Akel ME (2020). Experiential education in pharmacy curriculum: the Lebanese International University Model. Pharmacy (Basel).

[74] Order of Pharmacists of Lebanon (OPL). Guide to Self Assessment of Pharmacy Schools. 2018; Available from: https://opl.org.lb/newdesign/pdf/Pharmacy%20Programs%20Accreditation%20-%20Full%20Guide.pdf.

[75] Hallit S (2019). Good pharmacy practice standardized for community pharmacists: the Lebanese order of pharmacists initiative. J Res Pharm Pract.

[76] Badro DA (2020). Good pharmacy practice assessment among community pharmacies in Lebanon. Pharm Pract (Granada).

[77] Sacre H, Al-Worafi Y (2020). Drug safety in Lebanon. Drug safety in developing countries: achievements and challenges.

[78] Hallit S (2018). Medication safety knowledge, attitude, and practice among hospital pharmacists in Lebanon. J Eval Clin Pract.

[79] Hajj A (2018). Medication safety knowledge, attitudes and practices among community pharmacists in Lebanon. Curr Med Res Opin.

[80] Hajj A et al. Prescription and dispensing guidelines in Lebanon: initiative of the Order of Pharmacists of Lebanon*.* J Pharm Policy Pract. 2020; 13(70). 10.1186/s40545-020-00273-9 PMC7644285 33292614

[81] Mouhtadi BB (2018). Physician-community pharmacist collaborative care in diabetes management: a pilot study. J Drug Assess.

[82] Iskandar K (2017). Community pharmacy in Lebanon: a societal perspective. Pharm Pract (Granada).

[83] Tawil S (2020). Patients’ perceptions regarding pharmacists’ healthcare services: the case of Lebanon. J Pharm Pract Res.

[84] Hallit S, Selwan CA, Salameh P (2020). Primary health care policy and vision for community pharmacy and pharmacists in Lebanon. Pharm Pract (Granada).

[85] Soubra R (2021). Health seeking behaviour among Lebanese population: a highlight on seeking care from pharmacists. Eur J Gen Pract.

[86] Hallit S (2019). Emphasizing the role of pharmacist as a researcher: the Lebanese order of pharmacists' perspective. J Res Pharm Pract.

[87] Hallit S, Sacre H, Salameh P (2019). Role of a professional organization in promoting and conducting research: the Lebanese Order of Pharmacists' experience. Int J Pharm Pract.

